# Tigecycline-resistant *Escherichia coli* ST761 carrying *tet*(X4) in a pig farm, China

**DOI:** 10.3389/fmicb.2022.967313

**Published:** 2022-08-09

**Authors:** Jing Wang, Meng-Jun Lu, Zhen-Yu Wang, Yue Jiang, Han Wu, Zhi-Ming Pan, Xinan Jiao

**Affiliations:** ^1^Jiangsu Key Laboratory of Zoonosis, Jiangsu Co-Innovation Center for Prevention and Control of Important Animal Infectious Diseases and Zoonoses, Yangzhou University, Yangzhou, China; ^2^Key Laboratory of Prevention and Control of Biological Hazard Factors (Animal Origin) for Agrifood Safety and Quality, Ministry of Agriculture of China, Yangzhou University, Yangzhou, China

**Keywords:** plasmids, ST761, tigecycline resistance, *tet*(X4), *Escherichia coli*

## Abstract

This study aimed to investigate the prevalence and characterization of *tet*(X4) in *Escherichia coli* isolates from a pig farm in Shanghai, China, and to elucidate *tet*(X4) dissemination mechanism in this swine farm. Forty-nine (80.33%) *E. coli* strains were isolated from 61 samples from a pig farm and were screened for the presence of *tet*(X). Among them, six (12.24%) strains were positive for *tet*(X4) and exhibited resistance to tigecycline (MIC ≥ 16 mg/L). They were further sequenced by Illumina Hiseq. Six *tet*(X4)-positive strains belonged to ST761 with identical resistance genes, resistance profiles, plasmid replicons, and cgMLST type except that additional ColE10 plasmid was present in isolate SH21PTE35. Isolate SH21PTE31, as a representative ST761 *E. coli* strain, was further sequenced using Nanopore MinION. The *tet*(X4) in SH21PTE31 was located on IncFIA18/IncFIB(K)/IncX1 hybrid plasmid pYUSHP31-1, highly similar to other *tet*(X4)-carrying IncFIA18/IncFIB(K)/IncX1 plasmids from ST761 *E. coli* and other *E. coli* lineages in China. These IncFIA18/IncFIB(K)/IncX1 plasmids shared closely related multidrug resistance regions, and could reorganize, acquire or lose resistance modules mediated by mobile elements such as IS*CR2* and IS*26*. Phylogenetic analysis were performed including all *tet*(X4)-positive isolates obtained in this pig farm combined with 43 *tet*(X4)-positive *E. coli* from pigs, cow, pork, wastewater, and patients with the same ST from NCBI. The 50 *tet*(X4)-carrying *E. coli* ST761 isolates from different areas in China shared a close phylogenetic relationship (0-49 SNPs). In conclusion, clonal transmission of *tet*(X4)-positive *E. coli* ST761 has occurred in this swine farm. *E. coli* ST761 has the potential to become a high-risk clone for *tet*(X4) dissemination in China.

## Introduction

Tigecycline is considered as a last-resort antimicrobial agent to treat serious infections caused by multidrug-resistant bacteria, particularly carbapenem-resistant *Enterobacteriaceae* (Yaghoubi et al., 2021). However, the recent identification of novel plasmid-borne tigecycline resistance genes *tet*(X3) in *Acinetobacter baumannii* and *tet*(X4) in *Escherichia coli* from animals in China significantly impairs the clinical efficacy of tigecycline (He et al., 2019). Thus far, *tet*(X) and its variants [*tet*(X1)∼*tet*(X47)] have been identified in Gram-negative pathogens and encode flavin-dependent monooxygenase that modify tigecycline (Aminov, 2021; Li R. et al., 2021; Umar et al., 2021; Zhang et al., 2021). Among them, the mobile *tet*(X4) gene has been increasingly identified in *E. coli* from various sources including food-producing animals, wild birds, food products, humans, and the environment, mainly in China (He et al., 2019; Fang et al., 2020; Li et al., 2020; Li Y. et al., 2021; Dong et al., 2022; Liu et al., 2022). It has sporadically reported in countries outside of China, e.g., Singapore, Pakistan, Vietnam, United Kingdom, and Norway (Ding et al., 2020; Marathe et al., 2021; Mohsin et al., 2021; Dao et al., 2022; Martelli et al., 2022). The *tet*(X4) has subsequently detected in various *Enterobacteriaceae* species, such as *Proteus*, *A. baumannii*, *Aeromonas caviae*, *Citrobacter freundii*, *Enterobacter cloacae*, *E. hormaechei*, *Klebsiella pneumoniae*, and *Shewanella xiamenensis* (Chen et al., 2019; He et al., 2019; Zeng et al., 2021; Dao et al., 2022; Li et al., 2022; Wu et al., 2022; Zhai et al., 2022).

Although tigecycline is not applied in livestock, the *tet*(X4) gene and tigecycline resistance are frequently described in *E. coli* from food-producing animals (mainly pigs) in China (He et al., 2019; Fang et al., 2020; Li Y. et al., 2021; Liu et al., 2022). The heavy use of tetracyclines in animal production might facilitate the emergence and spread of *tet*(X) in livestock (He et al., 2019). In addition, conjugative/mobilizable plasmids and mobile elements play an essential role in the dissemination of *tet*(X4) in Enterobacteriaceae (Aminov, 2021). In this study, we aimed to investigate the prevalence and characterization of *tet*(X4) in *E. coli* isolates from one pig farm in Shanghai, China, to provide insights into the spread of *tet*(X4) in this swine farm.

## Materials and methods

### Sample collection and *tet*(X) detection

On 15 July 2021, 61 non-duplicate samples from pig feces (*n* = 41) and pig feed (*n* = 20) were collected from a pig farm in Shanghai, China. Samples were incubated in LB broth for 18∼24 h and then cultured on the MacConkey agar with and without 2 mg/L tigecycline. One *E. coli* isolate per plate was selected and identified by 16S rRNA gene sequencing (Kim et al., 2010). The presence of *tet*(X) were detected by PCR and sequencing (Wang et al., 2019).

### Antimicrobial susceptibility testing

The MICs of tigecycline were determined in all *E. coli* strains using the broth microdilution method and interpreted according to EUCAST clinical breakpoint (MIC ≥ 1 mg/L)^1^. The *tet*(X4)-positive isolates were further tested susceptibility to other 13 antimicrobial agents including ampicillin, cefotaxime, meropenem, gentamicin, amikacin, streptomycin, tetracycline, chloramphenicol, florfenicol, nalidixic acid, ciprofloxacin, colistin, and sulfamethoazole/trimethoprim by using the broth microdilution method. The results were interpreted according to Clinical Laboratory Standards Institute (CLSI) M100, 30th edition. Florfenicol (> 16 mg/L) and streptomycin (> 16 mg/L) were interpreted according to the epidemiological cut-off values for *E. coli* set by EUCAST (see Text Footnote 1). The *E. coli* strain ATCC 25922 was used for quality control.

### Conjugation experiments

Conjugation experiments were conducted according to a previously described protocol (Chen et al., 2007) using *E. coli* C600 (streptomycin-resistant) as the recipient strain. Transconjugants were selected on MacConkey agar plates supplemented with 2 mg/L tigecycline and 3,000 mg/L streptomycin.

### Whole genome sequencing and analysis

The *tet*(X4)-positive *E. coli* strains were sequenced on the Illumina Hiseq platform, and the quality-trimmed raw sequence data were assembled into contigs using SPAdes v.3.8.2 with -careful and -cov cut-off auto options. One representative *E. coli* isolate SH21PTE31 was sequenced using Nanopore MinION, assembling with Unicycler version 0.4.9. The genome sequences of them were analyzed multilocus sequence typing (MLST), resistance genes, and plasmid replicons by using the Center for Genomic Epidemiology (CGE) pipeline^2^. The *tet*(X4)-carrying plasmid pYUSHP31-1 in strain SH21PTE31 was analyzed by ISfinder^3^, BLAST^4^ and the Gene Construction Kit 4.5 (Textco BioSoftware, Inc., Raleigh, NC, United States). pYUSHP31-1 was compared with other similar plasmids using BLASTn and BRIG.

### Phylogenetic analysis of *tet*(X4)-Positive ST761 *Escherichia coli* strains

The genome sequences of 43 *tet*(X4)-positive ST761 *E. coli* strains in the NCBI database were downloaded (data collected on July 7th, 2022) (Supplementary Table 1). The phylogenetic tree of all the *tet*(X4)-carrying ST761 *E. coli* strains obtained from this pig farm and NCBI was constructed using Parsnp^5^ and visualized by iTOL^6^. Core genome MLST (cgMLST) profiles based on 2,513 alleles were analyzed using cgMLSTFinder 1.2^7^.

### Nucleotide sequence accession number

The whole genome sequences of *tet*(X4)-positive *E. coli* isolates have been deposited in the GenBank under accession number PRJNA836295.

## Results and discussion

### Characterization of *tet*(X4)-positive *Escherichia coli* isolates

A total of 49 *E. coli* strains were obtained from 61 samples. Among them, six strains (12.24%) from different fecal samples were positive for *tet*(X4), including five strains isolated under selection with tigecycline and one strain isolate without selection. The *tet*(X4)-positive isolates exhibited resistance to tigecycline (MIC ≥ 16 mg/L), and the remaining isolates showed susceptibility to tigecycline with MICs of 0.125 to 0.5 mg/L. These *tet*(X4)-positive isolates were also resistant to ampicillin, tetracycline, chloramphenicol, florfenicol, and sulfamethoazole/trimethoprim, but susceptible to cefotaxime, meropenem, gentamicin, amikacin, streptomycin, colistin, nalidixic acid, and ciprofloxacin (Supplementary Table 2). However, all tigecycline-resistant isolates failed to transfer *tet*(X4) to *E. coli* C600 *via* conjugation.

**FIGURE 1:**
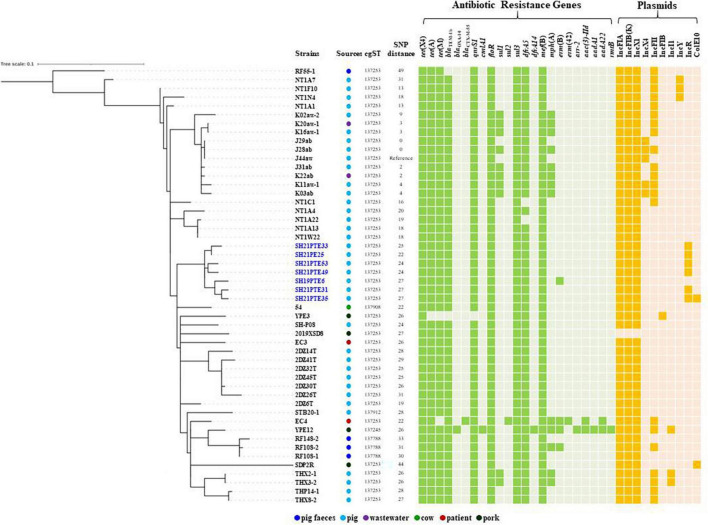
The maximum likelihood tree of *tet*(X4)-positive *E. coli* ST761 isolates in this study compared with *tet*(X4)-positive *E. coli* ST761 isolates from NCBI based on cgSNP analysis. Antibiotic resistance genes and plasmid replicons with >95% sequence homology and >60% coverage are shown. The isolates obtained in this study and in the same pig farm were indicated in blue.

The draft genome sequences of six *tet*(X4)-positive *E. coli* strains were obtained by Illumina (Supplementary Table 3). All six *tet*(X4)-positive *E. coli* strains belonged to ST761 with identical resistance genes [*bla*_*TEM–*1_, *tet*(A), *tet*(M), *floR*, *qnrS1*, *sul3*, *dfrA5* and *mef*(B)] and plasmid replicons [IncFIA, IncFIB(K), IncX1, IncR], except that additional ColE10 plasmid was present in isolate SH21PTE35 (Figure 1).

### *tet*(X4)-Carrying plasmid pYUSHP31-1

**FIGURE 2:**
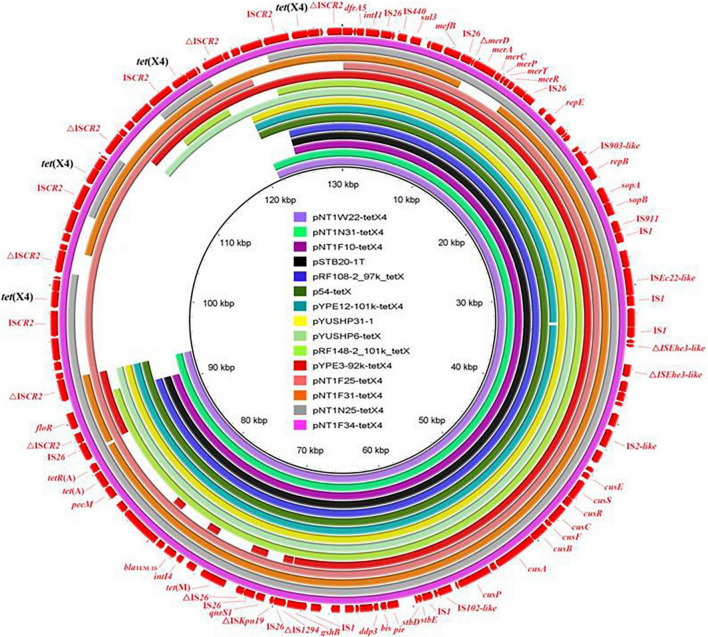
Sequence comparison of *tet*(X4)-carrying plasmid pYUSHP31-1 from *E. coli* isolate SH21PTE31 in this study with other similar IncFIA18/IncFIB(K)/IncX1 plasmids using BRIG. The reference sequence pNT1F34-tetX (CP075486) is indicated in red in the outer circle.

The complete sequences of isolate SH21PTE31, as a representative ST761 *E. coli* strain, was obtained. A total of 43,674 reads were obtained, and the sequencing data volume was approximately 1,000 Mbp. The minimal, maximum and average read lengths were 8,260 bp, 150,801 bp and 22,897.3 bp, respectively. The read length N50 of the total sequencing data were 28,637 bp. The isolate SH21PTE31 consisted of one chromosome (4,706,168 bp) and four plasmids (Supplementary Table 3). Among them, *tet*(X4) and another eight resistance genes were co-located on the largest plasmid, designated as pYUSHP31-1. This plasmid had a size of 104,163 bp, and belonged to the hybrid IncFIA18/IncFIB(K)/IncX1 plasmid. It was highly similar to our previously reported plasmid pYUSHP6-tetX (GenBank accession no. MW423609) from ST761 *E. coli* isolate SH19PTE6 collected from the same pig farm in 2019 (Wang et al., 2021), and also showed high identity (> 99.7%) to multiple *tet*(X4)-carrying IncFIA18/IncFIB(K)/IncX1 plasmids from ST761 *E. coli* strains in China, such as pNT1W22-tetX4 (pig, CP075470), pRF108-2_97k_tetX (pig, MT219820), pSTB20-1T (pig, CP050174), p54-tetX (cow, CP041286), pYPE12-101k-tetX4 (pork, CP041443), and pYPE3-92k-tetX4 (pork, CP041453) (Figure 2). Similar IncFIA18/IncFIB(K)/IncX1 plasmids harboring *tet*(X4) were also present among other *E. coli* lineages obtained from a pig farm in Jiangsu province, China (Li Y. et al., 2021), e.g., pNT1N31-tetX4 (ST716, CP075481), pNT1F25-tetX4 (ST1421, CP075471), pNT1F31-tetX4 (ST206, CP045188), pNT1N25-tetX4 (ST641, CP075485), and pNT1F34-tetX (ST10115, CP075486) (Figure 2), highlighting the importance role of horizontal transfer of plasmids in the *tet*(X4) dissemination between different bacteria.

**FIGURE 3:**
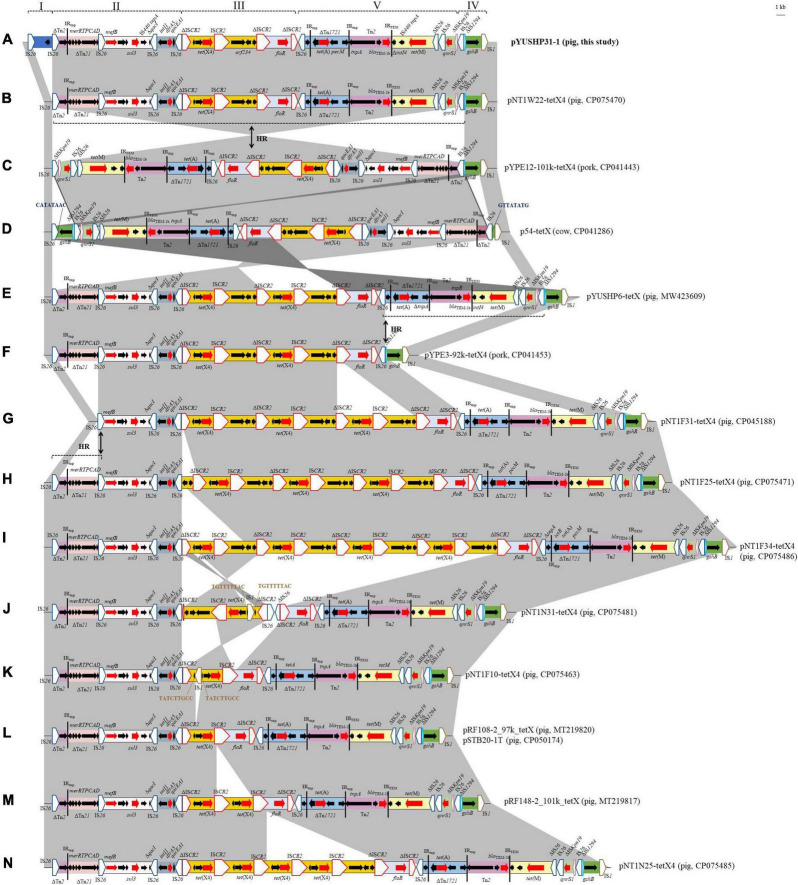
Genetic organization of the multidrug resistance region of plasmid pYUSHP31-1 and structural comparison with other IncFIA18/IncFIB(K)/IncX1 plasmids. I to IV indicate five regions bounded by IS26 or ISCR2 in pYUSHP31-1. The extents and directions of orientation of resistance genes (thick red arrow) and other genes are indicated by arrows. Regions with >99% identity are shaded in gray. 1 indicates a truncated gene or mobile element. Insertion sequences (ISs) are shown as boxes labeled with the IS name. Labeled vertical arrows with IS boxes denote the insertion position of IS elements. Direct repeats are indicated by arrows and sequences. Tall bars represent the 38-bp IR of transposons (Tn). Arrows labeled with “HR” and dotted lines indicate where homologous recombination could explain differences between structures.

As shown in Figure 3, these IncFIA18/IncFIB(K)/IncX1 plasmids shared closely related multidrug resistance regions (MRRs). The MRRs in all were bounded by one copy of IS*26* and IS*1*, respectively. The pYUSHP31-1 MRR (53,134 bp) contained nine resistance genes and consisted of five regions bounded by IS*26* or IS*CR2* (Figure 3A). The first of these (2,813 bp) comprised one copy of IS*26* and a putative open reading frame encoding recombinase family protein, which was absent in other similar plasmids.

The second part (∼14.8 kb) contained three resistance genes *mefB*, *sul3*, and *dfrA5*; four copies of IS*26* and incomplete transposon Tn*2* and Tn*21*. This fragment was also present in other IncFIA18/IncFIB(K)/IncX1 plasmids, but differed by 46-bp shorter (limited to pNT1N25-tetX4) or 126-bp longer Tn*2* except pYUSHP6-tetX (identical to pYUSHP31-1, obtained from the same pig farm); deletion of a 5,198-bp structure (IS*26*-ΔTn*2*-ΔTn*21*) in pNT1F31-tetX4 (Figure 3G).

The third region corresponded to the core *tet*(X4) structure [ΔIS*CR2-orf1-abh-tet*(X4)-IS*CR2-orf2-orf3-orf4*-ΔIS*CR2*] and downstream *floR*-ΔIS*CR2* module, as observed in other IncFIA18/IncFIB(K)/IncX1 plasmids with one to four copies of *tet*(X4) structure (Figures 3A–J). Compared with that of pYUSHP31-1, partial *tet*(X4) structure [ΔIS*CR2-orf1-abh-tet*(X4)-IS*CR2*] with varied copies was identified in plasmids pNT1F10-tetX4, pRF108-2_97k_tetX, pSTB20-1T, pRF148-2_101k_tetX, and NT1N25-tetX4 (Figures 3K–N); one copy of IS*1* was inserted into *orf1* within the *tet*(X4) structure with 9-bp direct repeats in plasmids pNT1F10-tetX4 and pNT1N31-tetX4, and the latter plasmid carried the *tet*(X4) fragment in the opposite orientation and additional two copies of IS*26* upstream of *floR*-ΔIS*CR2* module (Figure 3J). As previously described (Liu et al., 2022), IS*CR2* is associated with *tet*(X4) transmission by forming an rolling-cycle transposable unit, thus generating tandem copies of *tet*(X4)-harboring structures in different IncFIA18/IncFIB(K)/IncX1 plasmids.

The fourth segment (∼18.4 kb) included one copy of IS*26*, an incomplete Tn*1721* carrying tetracycline resistance gene *tet*(A) and an intact Tn*2* (*tnpA-tnpR-bla*_TEM–1b_), followed by 5,391-bp module [Δ*intI4*-IS*440 tnpA-tet*(M)-ΔIS*26*] and *qnrS1* structure (IS*26-qnrS1*-ΔIS*Kpn19*). This region was also found in other IncFIA18/IncFIB(K)/IncX1 plasmids with the same ΔIS*Kpn19*/IS*26* boundary except pYPE3-92k-tetX4 (Figure 3F). IS*26*-mediated homologous recombination could explain the loss or acquisition of this region.

The last segment comprising a 3,507-bp structure (IS*26*-ΔIS*1294-gshB*-IS*1*) was identical to segments in other plasmids except p54-tetX (Figure 3D). Insertion of an extra copy of IS*26* downstream of *gshB*, followed by homologous recombination between it and the first IS*26* of MRR, may explain the opposite location of an approximately 50.2-kb fragment within MRR in p54-tetX compared to pYUSHP31-1. Similar recombination between two IS*26* elements located in inverse orientations may also occur in pYPE12-101k-tetX4, leading to the presence of ∼47.8 kb fragment with the opposite orientation within MRR (Figure 3C).

These *tet*(X4)-carrying IncFIA18/IncFIB(K)/IncX1 plasmids may evolve from the same ancestor, and form variable but related MRRs by insertions, deletions, or rearrangements of different resistance modules mediated by mobile elements such as IS*26* and IS*CR2*.

### Phylogenomic analysis of *tet*(X4)-Positive ST761 *Escherichia coli* strains

*Escherichia coli* ST761 has been increasingly reported in different sources associated with *tet*(X4) in China, particularly from pigs (Supplementary Table 1). To further compare the genetic differences between *tet*(X4)-positive *E. coli* isolates of the same ST, we performed a phylogenomic analysis based on cgSNP. The results revealed a relatively close genetic relationship (0-49 SNPs) among 50 *tet*(X4)-positive ST761 *E. coli* isolates (Figure 1). Among them, cgST 137253 (*n* = 44) was the most prevalent type, and it contained two isolates from patients, two from wastewater, three from pork, and 37 from pigs including six strains obtained in this study and SH19PTE6 from the same pig farm (Figure 1). It indicates that clonal transmission has occurred in this swine farm. The plasmid replicons [IncFIA, IncFIB(K), IncX1] possibly associated with *tet*(X4) were present in all isolates, and the core resistance genes [*bla*_*TEM–*1_, *tet*(A), *tet*(M), *floR*, *qnrS1*, *sul3*, *dfrA5* and *mef*(B)] within *tet*(X4)-carrying IncFIA/IncFIB(K)/IncX1 plasmid pYUSHP31-1 were shared by 45 strains (Figure 1).

Although horizontal transfer mediated by plasmids (e.g., IncQ, and IncX1) and insertion sequences (e.g., IS*CR2*, IS*26*, and IS*1*) is the main mechanism for *tet*(X4) transmission (Aminov, 2021; Liu et al., 2022; Yu et al., 2022), clonal spread of *tet*(X4)-carrying strains, such as *E. coli* ST877, ST10, and ST48 clones is also responsible for *tet*(X4) dissemination between animals and humans (Cui et al., 2022). The *E. hormaechei* co-harboring *tet*(X4) and *bla*_*NDM*_ could also clonally spread from the slaughterhouse to the retail market (Li et al., 2022). *E. coli* ST761 isolates carrying *tet*(X4) has been detected in pigs, cow, pork, wastewater, and patients in different areas from China sharing a close phylogenetic relationship, suggesting that the ST761 lineage has the potential to be a successful clone to transfer *tet*(X4) and other resistance genes as well in China.

## Conclusion

Our findings suggest that *tet*(X4)-positive ST761 *E. coli* was the main reason for spread and persistence of *tet*(X4) in this pig farm. Importantly, *E. coli* ST761 has the potential to become a high-risk clone for *tet*(X4) dissemination in China. On the other hand, the *tet*(X4)-carrying IncFIA18/IncFIB(K)/IncX1 hybrid plasmids within ST761 *E. coli* lineage could reorganize, acquire or lose resistance modules mediated by mobile elements such as IS*CR2* and IS*26*. The horizontal transfer of similar IncFIA18/IncFIB(K)/IncX1 plasmids further facilitates the *tet*(X4) dissemination in distinct lineages.

## Data availability statement

The datasets presented in this study can be found in online repositories. The names of the repository/repositories and accession number(s) can be found in the article/Supplementary material.

## Author contributions

XJ and JW conceived the study. M-JL, HW, Z-YW, and YJ carried out the experiments. JW, Z-YW, and YJ analyzed the data. JW wrote the manuscript. Z-MP and XJ revised the manuscript. All authors read and approved the final manuscript.

## Footnotes

1
www.eucast.org

2
http://www.genomicepidemiology.org/

3
https://www-is.biotoul.fr/

4
https://blast.ncbi.nlm.nih.gov/Blast.cgi

5
https://harvest.readthedocs.io/en/latest/content/parsnp.html

6
https://itol.embl.de/

7
https://cge.food.dtu.dk/services/cgMLSTFinder/
