# Supplementary material for: Tigecycline-resistant Escherichia coli ST761 carrying tet(X4) in a pig farm, China
Source: Front Microbiol. 2022 Aug 9;13:967313. doi: 10.3389/fmicb.2022.967313 (PMC9396132; doi:10.3389/fmicb.2022.967313)
Supplement: Supplementary file 1 [file Data_Sheet_1.pdf]

Table S1 Detailed information of *tet*(X4)-positive ST761 *E. coli* strains

Strains	Source	Location	Year	Accession no.	***tet*(X4)-carrying plasmids**					Reference
					**plasmid**	**accession no.**	**size (bp)**	**plasmid replicons**	**resistance genes**	
THP14-1	pig	Fujian	14/07/2017	PRJNA757796						[1]
THX8-2	pig	Fujian	14/07/2017	PRJNA757796						[1]
THX2-1	pig	Fujian	14/07/2017	PRJNA757796						[1]
THX3-2	pig	Fujian	14/07/2017	PRJNA757796						[1]
STB20-1	pig	Jiangsu	08/05/2017	PRJNA548414	pSTB20-1T	CP050174	97,366	IncFIA/IncFIB(K)/IncX1	bla_{TEM-1b}/*tet*(A)/*tet*(X4)/*tet*(M)/ *floR*/*qnrS1*/*sul3*/*dfrA5*/*mef*(B)	[2]
EC3	patient	Zhejiang	2018	PRJNA595705	pEC3-tetX4		101,519	IncFIA/IncFIB(K)/IncX1	bla_{TEM-1b}/*tet*(A)/*tet*(X4)/*tet*(M)/ *floR*/*qnrS1*/*sul3*/*dfrA5*/*mef*(B)	[3]
EC4	patient	Zhejiang	2018	PRJNA595705	pEC4-tetX4		101,519	IncFIA/IncFIB(K)/IncX1	bla_{TEM-1b}/*tet*(A)/*tet*(X4)/*tet*(M)/ *floR*/*qnrS1*/*sul3*/*dfrA5*/*mef*(B)	[3]
NT1C1	pig	Jiangsu	2018	PRJNA663118						[4]
NT1A1	pig	Jiangsu	2018	PRJNA663118						[4]
NT1F10	pig	Jiangsu	2018	PRJNA663118	pNT1F10-tetX4	CP075463	97,500	IncFIA/IncFIB(K)/IncX1	bla_{TEM-1b}/*tet*(A)/*tet*(X4)/*tet*(M)/ *floR*/*qnrS1*/*sul3*/*dfrA5*/*mef*(B)	[4]
NT1N4	pig	Jiangsu	2018	PRJNA663118						[4]
NT1A7	pig	Jiangsu	2018	PRJNA663118						[4]
NT1A4	pig	Jiangsu	2018	PRJNA663118						[4]
NT1A22	pig	Jiangsu	2018	PRJNA663118						[4]
NT1A13	pig	Jiangsu	2018	PRJNA663118						[4]
NT1W22	pig	Jiangsu	2018	PRJNA663118	pNT1W22-tetX4	CP075470	101,476	IncFIA/IncFIB(K)/IncX1	bla_{TEM-1b}/*tet*(A)/*tet*(X4)/*tet*(M)/ *floR*/*qnrS1*/*sul3*/*dfrA5*/*mef*(B)	[4]
SH-P08	pig	Shanghai	2018	PRJNA625924						[5]
54	cow	China	2018	PRJNA551552	p54-tetX	CP041286	102,347	IncFIA/IncFIB(K)/IncX1	bla_{TEM-1b}/*tet*(A)/*tet*(X4)/*tet*(M)/ *floR*/*qnrS1*/*sul3*/*dfrA5*/*mef*(B)	NA
2019XSD8	pork	Shandong	2019	PRJNA532458						[6]
RF55-1	pig faeces	Jiangsu	05/2019	PRJNA612724						[7]

RF108-1	pig faeces	Nantong, Jiangsu	05/2019	PRJNA612741	pRF108-1_107k_tetX_flye		107,701	IncFIA/IncFIB(K)/IncX1	*bla*$_{TEM-1b}$/*tet*(A)/*tet*(X4)/*tet*(M)/*floR*/*qnrS1*/*sul3*/*dfrA5*/*mef*(B)	[7]
RF108-2	pig faeces	Nantong, Jiangsu	05/2019	PRJNA612742	pRF108-2_97k_tetX	MT219820	97,526	IncFIA/IncFIB(K)/IncX1	*bla*$_{TEM-1b}$/*tet*(A)/*tet*(X4)/*tet*(M)/*floR*/*qnrS1*/*sul3*/*dfrA5*/*mef*(B)	[7]
RF148-2	pig faeces	Jiangsu	05/2019	PRJNA612746	pRF148-2_101k_tetX	MT219817	101,373	IncFIA/IncFIB(K)/IncX1	*bla*$_{TEM-1b}$/*tet*(A)/*tet*(X4)/*tet*(M)/*floR*/*qnrS1*/*sul3*/*dfrA5*/*mef*(B)	[7]
YPE3	pork	Yangzhou, Jiangsu	06/2019	PRJNA553293	pYPE3-92k-tetX4	CP041453	92,973	IncFIA/IncFIB(K)/IncX1	*bla*$_{TEM-1b}$/*tet*(X4)/*floR*/*sul3*/*dfrA5*/*mef*(B)	[8]
YPE12	pork	Yangzhou, Jiangsu	06/2019	PRJNA553293	pYPE12-101k-tetX4	CP041443	101,987	IncFIA/IncFIB(K)/IncX1	*bla*$_{TEM-1b}$/*tet*(A)/*tet*(X4)/*tet*(M)/*floR*/*qnrS1*/*sul3*/*dfrA5*/*mef*(B)	[8]
SDP2R	pork	Shandong	2019	PRJNA665928						[9]
K02aw-2	pig	Hunan	2020	PRJNA689679						NA
K11aw-1	pig	Hunan	2020	PRJNA689679						NA
K16aw-1	pig	Hunan	2020	PRJNA689679						NA
K20aw-1	wastewater	Hunan	2020	PRJNA689679						NA
K22ab	wastewater	Hunan	2020	PRJNA689679						NA
K03ab	pig	Hunan	2020	PRJNA689679						NA
J28ab	pig	Hunan	2020	PRJNA689679						NA
J29ab	pig	Hunan	2020	PRJNA689679						NA
J31ab	pig	Hunan	2020	PRJNA689679						NA
J44aw	pig	Hunan	2020	PRJNA689679						NA
2DZ6T	pig	Qingdao, Shandong	2019	PRJNA800767						[10]
2DZ14T	pig	Qingdao, Shandong	2019	PRJNA800767						[10]
2DZ26T	pig	Qingdao, Shandong	2019	PRJNA800767						[10]
2DZ30T	pig	Qingdao, Shandong	2019	PRJNA800767	p2DZ30T		172,253	IncFIA/IncFIB(K)/IncX1	*bla*$_{TEM-1b}$/*tet*(A)/*tet*(X4)/*tet*(M)/*floR*/*qnrS1*/*sul3*/*dfrA5*/*mef*(B)	[10]

2DZ32T	pig	Qingdao, Shandong	2019	PRJNA800767						[10]
2DZ41T	pig	Qingdao, Shandong	2019	PRJNA800767						[10]
2DZ45T	pig	Qingdao, Shandong	2019	PRJNA800767						[10]
SH19PTE6*	pig	Shanghai	23/09/2019	PRJNA724799	pYUSHP6-tetX	MW423609	111,332	IncFIA/IncFIB(K)/IncX1	*bla*$_{TEM-1b}$/*tet*(A)/*tet*(X4)/*tet*(M)/*floR*/*qnrS1*/*sul3*/*dfrA5*/*mef*(B)	[11]
SH21PTE31	pig	Shanghai	15/07/2021	PRJNA836295	pYUSHP31-1	CP097182	104,163	IncFIA/IncFIB(K)/IncX1	*bla*$_{TEM-1b}$/*tet*(A)/*tet*(X4)/*tet*(M)/*floR*/*qnrS1*/*sul3*/*dfrA5*/*mef*(B)	this study
SH21PTE33	pig	Shanghai	15/07/2021	PRJNA836295						this study
SH21PTE35	pig	Shanghai	15/07/2021	PRJNA836295						this study
SH21PTE49	pig	Shanghai	15/07/2021	PRJNA836295						this study
SH21PTE53	pig	Shanghai	15/07/2021	PRJNA836295						this study
SH21PE25	pig	Shanghai	15/07/2021	PRJNA836295						this study

* indicates that it was obtained from the same pig farm described in this study in 2019; NA: not available.

References

[1] Sun, J., Chen, C., Cui, C.Y., Zhang, Y., Liu, X., Cui, Z.H., et al. (2019). Plasmid-encoded *tet*(X) genes that confer high-level tigecycline resistance in *Escherichia coli*. *Nat. Microbiol.* 4, 1457-1464. doi: 10.1038/s41564-019-0496-4.

[2] Fang, L.X., Chen, C., Yu, D.L., Sun, R.Y., Cui, C.Y., Chen, L., et al. (2019). Complete nucleotide sequence of a novel plasmid bearing the high-level tigecycline resistance gene *tet*(X4). *Antimicrob. Agents Chemother.* 63, e01373-19. doi: 10.1128/AAC.01373-19.

[3] Zhang, R., Dong, N., Shen, Z., Zeng, Y., Lu, J., Liu, C., et al. (2020). Epidemiological and phylogenetic analysis reveals *Flavobacteriaceae* as potential ancestral source of tigecycline resistance gene *tet*(X). *Nat. Commun*. 11, 4648. doi: 10.1038/s41467-020-18475-9.

[4] Li, Y., Wang, Q., Peng, K., Liu, Y., Xiao, X., Mohsin, M., and Wang, Z. (2021b). Distribution and genomic characterization of tigecycline-resistant *tet*(X4)-positive *Escherichia coli* of swine farm origin. *Microbial. Genomics*. 7, 000667. doi: 10.1099/mgen.0.000667.

[5] Sun, C., Cui, M., Zhang, S., Liu, D., Fu, B., Li, Z., et al. (2020). Genomic epidemiology of animal-derived tigecycline-resistant *Escherichia coli* across China reveals recent endemic plasmid-encoded *tet*(X4) gene. *Commun. Biol*. 3, 412. doi: 10.1038/s42003-020-01148-0.

[6] Bai, L., Du, P., Du, Y., Sun, H., Zhang, P., Wan, Y., et al. (2019). Detection of plasmid-mediated tigecycline-resistant gene *tet*(X4) in *Escherichia coli* from pork, Sichuan and Shandong Provinces, China, February 2019. *Euro Surveill*. 24, 1900340. doi: 10.2807/1560-7917.ES.2019.24.25.1900340.

[7] Li, R., Lu, X., Peng, K., Liu, Z., Li, Y., Liu, Y., et al. (2020). Deciphering the structural diversity and classification of the mobile tigecycline resistance gene *tet*(X)-bearing plasmidome among bacteria. *mSystems*, 5, e00134-20. doi: 10.1128/mSystems.00134-20.

[8] Li, R., Lu, X., Liu, Z., Liu, Y., Xiao, X., and Wang, Z. (2020). Rapid detection and characterization of *tet*(X4)-positive *Escherichia coli* strains with nanopore sequencing. *J. Antimicrob. Chemother.* 75, 1068-1070. doi: 10.1093/jac/dkz528.

[9] Li, R., Li, Y., Peng, K., Yin, Y., Liu, Y., He, T., et al. (2021). Comprehensive genomic investigation of tigecycline resistance gene *tet*(X4)-bearing strains expanding among different settings. *Microbiol. Spectr*. 9, e0163321. doi: 10.1128/spectrum.01633-21.

[10] Zhai, W., Wang, T., Yang, D., Zhang, Q., Liang, X., Liu, Z., et al. (2022). Clonal relationship of *tet*(X4)-positive *Escherichia coli* ST761 isolates between animals and humans. *J. Antimicrob. Chemother*. doi: 10.1093/jac/dkac175.

[11] Wang, J., Wu, H., Mei, C.Y., Wang, Y., Wang, Z.Y., Lu, M.J., et al. (2021). Multiple mechanisms of tigecycline resistance in *Enterobacteriaceae* from a pig farm, China. *Microbiol. Spectr*. 9, e0041621. doi: 10.1128/Spectrum.00416-21.

Table S2 MICs of tested antimicrobial agents against the *tet*(X4)-positive *Escherichia coli* strains in this study.

	SH21PTE31	SH21PTE33	SH21PTE35	SH21PTE49	SH21PTE53	SH21PE25
Sources	sow	sow	sow	fattening pig	fattening pig	boar
MIC (mg/L)						
Ampicillin	>128	>128	>128	>128	>128	>128
Cefotaxime	0.06	0.06	0.03	0.06	0.06	0.03
Meropenem	0.015	0.03	0.03	0.015	0.015	0.015
Gentamicin	2	2	2	1	1	1
Streptomycin	4	4	4	4	4	4
amikacin	1	2	2	1	1	1
Tetracycline	>128	>128	>128	>128	>128	>128
Tigecycline	16	16	16	16	32	16
Chloramphenicol	>128	>128	>128	>128	>128	>128
Florfenicol	>128	>128	>128	>128	>128	>128
Colistin	0.125	0.25	0.25	0.25	0.25	0.125
Nalidixic acid	16	8	8	16	8	8
Ciprofloxacin	0.25	0.125	0.25	0.25	0.25	0.25
Sulfamethoxazole/trimethoprim	64	64	64	64	64	64

Table S3 Data quality statistics obtained by Illumina

	**SH21PE25**	**SH21PTE31**	**SH21PTE35**	**SH21PTE33**	**SH21PTE49**	**SH21PTE53**
**contigs (⩾0 bp)**	194	188	192	199	188	181
**contigs (⩾1000 bp)**	106	112	110	108	108	108
**contigs (⩾5000 bp)**	74	76	77	75	76	75
**contigs (⩾10000 bp)**	66	68	68	66	68	67
**contigs (⩾25000 bp)**	52	51	52	50	52	52
**contigs (⩾50000 bp)**	25	25	26	24	25	25
**total length (⩾0 bp)**	4740793	4773199	4768965	4778050	4767255	4760345
**total length (⩾1000 bp)**	4702234	4738942	4732921	4739379	4731835	4728552
**total length (⩾5000 bp)**	4612970	4646037	4642049	4647207	4645020	4636890
**total length (⩾10000 bp)**	4556907	4589783	4576476	4585663	4589353	4581206
**total length (⩾25000 bp)**	4334873	4329647	4329420	4334729	4346363	4350108
**total length (⩾50000 bp)**	3291310	3318301	3325536	3323373	3300188	3303709
**contigs**	139	139	138	140	135	134
**largest contigs**	492139	491758	319906	492402	491759	416563
**Total length**	4723349	4756927	4750882	4759914	4749396	4745196
**GC(%)**	50.73	50.71	50.66	50.70	50.70	50.70
**N50**	100951	103257	103257	130346	101627	103984
**N75**	44818	44818	45263	44719	44818	44818
**L50**	12	12	13	11	12	12
**L75**	31	31	31	30	31	31
**N's per 100 kbp**	5.40	2.94	3.45	7.50	3.18	7.61

All statistics are based on contigs of size ≥500 bp, unless otherwise noted e.g., "contigs (≥ 0 bp)" and "total

length (≥ 0 bp)" include all contigs.

Table S3 Whole genome of ST761 *Escherichia coli* strain SH21PTE31 in this study.

	**Size (bp)**	**Resistance genes**	**Plasmid replicon**
SH21PTE31			
chromosome	4,706,168	-	
pYUSHP31-1	104,163	bla_{TEM-1b}, *tet*(X4), *tet*(A), *tet*(M), *qnrS1*, *floR*, *sul3*, *dfrA5*, *mef*(B)	IncFIA18/IncFIB(K)/IncX1
pYUSHP31-2	64,527	-	IncFIA/IncR
pYUSHP31-3	4,145	-	NT
pYUSHP31-4	4,072	-	NT

-, Not found; NT, not typable.
